# Use of Warfarin or Direct Oral Anticoagulants and Risk of Prostate Cancer in PCBaSe: A Nationwide Case-Control Study

**DOI:** 10.3389/fonc.2020.571838

**Published:** 2020-10-08

**Authors:** Jonathan Parker, Danielle Crawley, Hans Garmo, Bertil Lindahl, Johan Styrke, Jan Adolfsson, Mats Lambe, Pär Stattin, Mieke Van Hemelrijck, Kerri Beckmann

**Affiliations:** ^1^Translational Oncology and Urology Research Group, School of Cancer and Pharmaceutical Sciences, King's College London, London, United Kingdom; ^2^Regional Cancer Centre Uppsala Orebro, Uppsala, Sweden; ^3^Clinical Research Centre, Uppsala University Hospital, Uppsala, Sweden; ^4^Department of Surgical and Perioperative Sciences, Umeå University, Umeå, Sweden; ^5^Department of Clinical Science, Intervention and Technology, Karolinska Institutet, Stockholm, Sweden; ^6^Department of Medical Epidemiology and Biostatistics, Karolinska Institutet, Stockholm, Sweden; ^7^Department of Surgical Sciences, Uppsala University Hospital, Uppsala, Sweden; ^8^Cancer Research Institute, School of Health Sciences, University of South Australia, Adelaide, SA, Australia

**Keywords:** prostate cancer, warfarin, direct anticoagulant agents, detection bias, case-control study

## Abstract

Existing literature examining warfarin's association with prostate cancer (PCa) risk provides conflicting results, while the association with direct oral anticoagulants (DOACs) has not yet been studied. We investigated the association of warfarin and DOAC use on PCa risk among men within the population-based Prostate Cancer database Sweden (PCBaSe), using a case-control design. The study population included PCa cases diagnosed 2014–2016 and five age-matched PCa-free controls. Conditional logistic regression was used to estimate odds ratios (ORs) with 95% confidence intervals (CI) for PCa associated with warfarin and DOAC use, adjusted for marital status, education level, other drug use, and comorbidities. Among 31,591 cases and 156,802 controls, there were 18,522 (9.8%) warfarin and 4,455 (2.4%) DOAC users. Warfarin ever-use was associated with reduced risk of PCa overall (OR 0.92 95% CI 0.88–0.96) as were both past and current use. DOAC use was not associated with PCa risk. For some warfarin exposures, decreased risk was observed for unfavorable PCa (high risk/locally advanced/distant metastatic) but not with favorable PCa (low/intermediate risk). Increased risk of favorable PCa was observed for men whose initial warfarin exposure occurred in the 12 month period before diagnosis (OR 1.39; 95% CI 1.13–1.70). Our findings are consistent with previous publications reporting decreased PCa risk with warfarin exposure. Increased risk of favorable PCa suggests detection bias due to increased prostate specific antigen testing when starting on warfarin. Decreased overall PCa risk could reflect bias due to reduced biopsy rates among long-term warfarin users.

## Introduction

Prostate cancer (PCa) is the second most common cancer in men with an estimated 1.3 million incident cases diagnosed in 2018 (1). Established risk factors for PCa include age, ethnicity, and family history (2).

Anticoagulants such as vitamin K antagonists (VKAs) are commonly prescribed to patients at risk of, or with a history of, thromboembolic events, atrial fibrillation/flutter and mechanical heart valve (3). Many of these men will also be diagnosed with PCa. A possible protective effect of VKAs on the development of PCa was first reported in 2000 (4) prompting an interest in the possible anti-PCa effect of VKAs, in particular, warfarin (5–12). However, to date evidence is conflicting, with a recent meta-analysis finding no association between VKAs and PCa risk (9). Detection bias due to reluctance to perform prostate biopsies among men on chronic oral anticoagulation therapy has been suggested as a possible explanation for decreased risk of Pca in men on warfarin (13).

Direct oral anticoagulants (DOACs) directly target factors Xa or IIa of the coagulation cascade and reduce the risk of venous thromboembolism, acute ischemic stroke, and bleeding events, as effectively as warfarin (14–17). In contrast to warfarin, DOACs do not require monitoring, and the use of DOACs been increasing in the past decade (18). Given this increase, the use of DOACs in relation to PCa risk is also of interest.

The aim of this study was to investigate the association between warfarin use, DOAC use, and PCa risk using population-based data in Sweden.

## Methods

### Data Sources

A nationwide case-control study was performed, comparing warfarin use, and DOAC use in patients with PCa (cases) to men in the general population without PCa (controls).

We used data from Prostate Cancer database Sweden (PCBaSe) 4.0, which includes all men recorded in the National Prostate Cancer Register (NPCR) of Sweden diagnosed with PCa since 1998. In PCBaSe, the NPCR is linked to the Swedish Cancer Register, Cause of Death Register, Prescribed Drug Register, National Patient Register, and Longitudinal Integration Database for Health Insurance and Labor Market Studies (LISA), by use of the Swedish Personal Identity Number (19, 20). PCBaSe contains information on date of PCa diagnosis, age at diagnosis, primary treatment, tumor stage and grade, prostate-specific antigen (PSA) levels at time of diagnosis, hospital admissions, prescribed medications and date and cause of death (21, 22). In PCBaSe 4.0, all men diagnosed with PCa are matched with five PCa-free men residing in the same county and with the same year of birth, to serve as controls. Random selection of controls, with replacement, was undertaken by Statistics Sweden. Controls were verified through the Swedish Cancer Register as PCa-free at the time their respective case was diagnosed. For the purpose of the present study, cases included all men diagnosed with PCa between 2014 and 2016. Controls were assigned their respective case's diagnosis date for analyses. No exclusion criteria were applied.

### Anticoagulant Use

Prior exposure to warfarin or DOACs was determined from anatomical therapeutic codes (ATCs) for filled prescriptions, recorded in the Prescribed Drug Register. The earliest exposure date for all prescriptions, including warfarin or DOACs was July 1st 2005, coinciding with start of the NPDR. Ever-use was defined as two or more filled prescriptions for warfarin (ATC B01AA03) or for DOACs (ATC: B01AF01—rivaroxaban, B01AF02—apixaban, B01AF03—edoxaban, B01AF07—dabigatran extexilate). Edoxaban was not prescribed in this study population. Past use of warfarin or DOACs was defined as two or more filled prescriptions but no prescriptions within 6 months of diagnosis. Current use of warfarin or DOACs was defined as two or more prescriptions with at least one being filled within 6 months of diagnosis. Recent initiation was defined as a first warfarin or DOAC prescription date (i.e., first-time prescription) within 12 months prior to PCa diagnosis, providing at least two prescriptions had been filled.

### Covariates

Comorbidity burden was assessed using the Charlson Comorbidity index (CCI) (23) based on hospital discharge information from the National Patient Register up to 10 years prior to the diagnosis date of PCa. In addition, diagnostic and procedural ICD-10-codes were retrieved on mechanical heart valve (Z95.2, FKD00, FMD00), pulmonary embolism (I26), portal vein thrombosis/other venous embolism and thrombosis (I81/I82), atrial fibrillation and flutter (I48). Any past exposure to other potential confounding medications was determined using ATCs for filled prescriptions in the Prescribed Drug Registry, including antidiabetic (T2DM) drugs (ATCs: A10A, A10AC, A10AD, A10AE, A10AF, A10BA, A10BB), cardiac drugs (ATCs: C07, C08, C09A, C09B, C09C, C09D), and statins (ATC: C10. Socioeconomic factors (civil status and highest education level) were extracted from the Longitudinal Integrated Database for Health Insurance and Labor market Studies (LISA).

### PCa Classification and Characteristics

Additional information on PSA level at diagnosis, Gleason Grade Group (24), primary treatment, mode of detection and risk category was obtained from the NPCR. PCa risk categories in the NPCR are a modified version of the National Comprehensive Cancer Network guidelines: low-risk (T1–2, Gleason score 2–6 and PSA < 10 ng/ml): intermediate-risk (T1–2, Gleason score 7 and/or PSA 10 to < 20 ng/ml); high risk (T3 and/or Gleason score 8–10 and/or PSA 20 to < 50 ng/ml); locally advanced [T4 and/or N1 and/or PSA 50 to < 100 ng/ml in the absence of distant metastases (M0 or Mx)]; distant metastases (M1 and/or PSA ≥ 100 ng/ml) (22). For subgroup analyses we classified PCa cases as having favorable PCa (low and intermediate risk PCa) and unfavorable PCa (high risk, locally advanced and distant metastatic PCa) at diagnosis.

### Analysis

Multivariable conditional logistic regression was used to estimate odds ratios (ORs) and 95% confidence intervals (CI) between use of warfarin or DOACs, and PCa risk. Since less than one percent of the study cohort had been exposed to both drugs, exposure to warfarin, and DOACs were considered in separate analysis (without mutual adjustment).

Models were adjusted for the following factors: previous exposure to T2DM-drugs, cardiac drugs, and statins; CCI at diagnosis (0, 1, 2, 3, ≥ 4); highest achieved educational level (≤9, 9–12, > 12 years), and marital status (married, divorced, widower, unmarried). Use of T2DM-drugs, cardiac drugs and statins were included due to potential associations with risk of PCa (25–27). Since cases and controls were matched on age and county of residence these variables were not included as a covariate in regression models. Further subgroup analyses (adjusted) were undertaken to evaluate if use of warfarin or DOACs was associated with more or less favorable PCa.

All analysis was performed using STATA version 15, StataCorp Limited Liability Company (Texas, USA).

### Ethics

The study was approved by the Ethical Review Board, Umea, Sweden.

## Results

The study population included 31,591 cases and 156,802 controls. The proportion of men who had been exposed to warfarin and DOACs were similar for cases and controls (warfarin 9.2 vs. 10.0% and DOACs 2.3 vs. 2.4%, respectively). Prior exposure to T2DM medication or statins was lower among cases than controls (Table 1). The majority of both cases (78.4%) and controls (77.0%) had no documented comorbidities. Among PCa cases, 45% had PSA levels of > 10–20 ng/ml at diagnosis whilst 60% were recorded as Gleason grade group 1 or 2 (Table 2). The majority of cases had favorable PCa.

**Table 1:** Characteristics of prostate cancer cases diagnosed 2014–2016 and their matched controls.

**Characteristic**	**Cases**	**Controls**
	**(*n =* 31,591)**	**(*n =* 156,802)**
**Age (Years)**		
Median (IQR)	70 (64–76)	70 (64–76)
<60	3,978 (12.6%)	19,724 (12.6%)
60–69	12,581 (39.8%)	62,094 (39.6%)
70–79	10,964 (34.7%)	54,770 (34.9%)
>79	4,068 (12.9%)	20,214 (12.9%)
**Anticoagulant use^a^^,^^b^**		
Warfarin ever-use	2,915 (9.2%)	15,607 (10.0%)
Doac ever-use	734 (2.3%)	3,721 (2.4%)
**Other medications^b^**		
T2dm drugs	3,461 (11.0%)	21,554 (13.8%)
Cardiac drugs	17,532 (55.5%)	87,102 (55.6%)
Statins	10,370 (32.8%)	54,359 (34.7%)
**Medical history^b^**		
Mechanical heart valve	180 (0.6%)	1,004 (0.6%)
Pulmonary embolism	548 (1.7%)	2,412 (1.5%)
Pvt/vte	212 (0.7%)	1,027 (0.7%)
Atrial fibrillation	3,271 (10.4%)	17,887 (11.4%)
**Charlson comorbidity index^b^**		
0	24,769 (78.4%)	120,671 (77.0%)
1	3,114 (9.9%)	16,181 (10.3%)
2	2,114 (6.7%)	10,394 (6.6%)
3	833 (2.6%)	4,498 (2.9%)
≥4	761 (2.4%)	5,058 (3.2%)
**Highest education achieved (Years)**		
≤ 9	9,356 (29.6%)	49,477 (31.6%)
>9–12	13,099 (41.5%)	64,304 (41.0%)
>12	8,931 (28.3%)	41,145 (26.2%)
Unknown	205 (0.7%)	1,879 (1.2%)
**Marital status**		
Married	20,240 (64.1%)	93,801 (59.8%)
Divorced	4,968 (15.7%)	27,002 (17.2%)
Widower	2,228 (7.1%)	11,257 (7.2%)
Unmarried	4,144 (13.1%)	24,735 (15.8%)
Unknown	11 (0.0%)	7 (0.0%)

*IQR, interquartile range; DOAC, Direct Oral Anticoagulant; T2DM, Type 2 Diabetes Mellitus; PVT, portal vein thrombosis; VTE, venous thromboembolism or venous embolism and thrombosis*.

a *defined as two or more filled prescriptions*.

b *defined by ICD codes*.

**Table 2:** Cancer characteristics in prostate cancer cases in PCBaSe 4.0 diagnosed 2014–2016.

	**Cases (*n =* 3,591)**
**PSA level (ng/ml)^a^**	
≤ 4	4,298 (13.6%)
>4–10	6,947 (22.0%)
>10–20	14,176 (44.9%)
>20	5,445 (17.2%)
Unknown	725 (2.3%)
**Gleason grade group**	
Grade Group 1	11,381 (36.0%)
Grade Group 2	8,286 (26.2%)
Grade Group 3	4,166 (13.2%)
Grade Group 4	2,883 (9.1%)
Grade Group 5	3,995 (12.7%)
Unknown	880 (2.8%)
**Mode of detection**	
Health check-up	16,437 (52.0%)
Lower urinary tract symptoms	9,147 (29.0%)
Other symptoms	4,973 (15.7%)
Unknown	1,034 (3.3%)
**Risk group**	
**Favorable^b^**	19,222 (60.9%)
Low risk	8,607 (27.3%)
Intermediate risk	10,615 (33.6%)
**Unfavorable^c^**	6,252 (36.5%)
High risk	6,117 (19.4%)
Locally advanced	1,694 (5.4%)
Distant metastases	3,731 (11.8%)
Unknown	827 (2.6%)

*PSA, prostate specific antigen*.

a *PSA level at time of diagnosis*.

b *favorable—low risk (T1–2, Gleason score 2–6 and PSA < 10 ng/ml) and intermediate risk (T1–2, Gleason score 7 and/or PSA 10 to < 20 ng/ml)*.

c *unfavorable—high risk (T3 and/or Gleason score 8–10 and/or PSA 20 to < 50 ng/ml), locally advanced (T4 and/or N1 and/or PSA 50 to < 100 ng/ml in the absence of distant metastases (M0 or Mx) and distant metastases (M1 and/or PSA ≥ 100 ng/ml and above)*.

Unadjusted and adjusted analysis of overall PCa risk are presented in Table 3. Compared with men never exposed to warfarin, those ever exposed had a decreased risk of PCa overall, in both unadjusted (OR 0.92 95% CI 0.88–0.96) and adjusted (OR 0.92; 95% CI 0.88–0.96) models.

**Table 3:** Odds ratios for overall prostate cancer risk.

		**Unadjusted^a^**			**Adjusted^a^^,^^b^**	
	**OR**	**95% CI**	***P*-value**	**OR**	**95% CI**	***P*-value**
Warfarin never exposure	1.00	(*Ref*)		1.00	(*Ref*)	
Warfarin ever exposure^c^	0.92	0.88–0.96	<0.001	0.92	0.88–0.96	<0.001
**Other medications^d^**						
T2dm drugs	0.77	0.74–0.80	<0.001	0.80	0.77–0.83	<0.001
Cardiac drugs	1.00	0.97–1.02	0.925	1.07	1.04–1.10	<0.001
Statins	0.92	0.89–0.94	<0.001	0.94	0.92–0.97	<0.001
**Charlson comorbidity** **index^d^**						
0	1.00	(*Ref*)		1.00	(*Ref*)	
1	0.93	0.90–0.97	0.001	0.97	0.93–1.01	0.168
2	0.98	0.94–1.03	0.506	1.03	0.98–1.08	0.251
3	0.89	0.83–0.96	0.004	0.98	0.90–1.06	0.563
≥4	0.73	0.67–0.79	<0.001	0.81	0.75–0.88	<0.001
**Education level**						
≤ 9 years	1.00	(*Ref*)		1.00	(*Ref*)	
10–12 years	1.08	1.05–1.12	<0.001	1.07	1.04–1.10	<0.001
>12 years	1.16	1.12–1.20	<0.001	1.12	1.08–1.15	<0.001
**Marital status**						
Married	1.00	(*Ref*)		1.00	(*Ref*)	
Divorced	0.85	0.82–0.88	<0.001	0.86	0.83–0.89	<0.001
Widower	0.93	0.88–0.97	0.003	0.94	0.89–0.99	0.013
Unmarried	0.77	0.74–0.80	<0.001	0.79	0.76–0.82	<0.001

*OR, odds ratio; CI, confidence intervals; T2DM, Type II Diabetes Mellitus*.

a *matched on age and county of residency*.

b *adjusted for all variables presented in the table*.

c *defined as two or more filled prescriptions*.

d *defined ICD codes*.

Multivariable adjusted analysis for timing of exposure is presented in Table 4. We observed a slightly elevated non-significant difference in overall PCa risk among men whose initial exposure to warfarin occurred within 12 months of PCa diagnosis compared to those never exposed (OR 1.15; 95% CI 0.98–1.33). However, if first recorded exposure was more than 12 months before PCa diagnosis (majority of warfarin-users) the risk of PCa was lower (OR 0.91; 95% CI 0.87–0.95). Both past and current use of warfarin were associated with decreased risk of PCa (OR 0.89; 95% CI 0.82–0.96 and 0.94; 95% CI 0.89–0.98, respectively).

**Table 4:** Odds ratios for overall risk of prostate cancer associated with warfarin or directoral anticoagulants (DOAC) according to time of exposure.

	**Cases**	**Controls**	**OR**	**Adjusted OR^a^**	***p*-Value**
	**no. (%)**	**no. (%)**		**% CI**	
**Warfarin**					
No exposure	28,676 (90.8%)	141,195 (90.1%)	1.00	(*Ref*)	
Ever exposure^b^	2,915 (9.2%)	15,607 (10.0%)	0.92	0.88–0.96	<0.001
Past-use^c^	744 (2.4%)	4,165 (2.7%)	0.89	0.82–0.96	0.003
Current-use^d^	2171 (6.9%)	11,442 (7.3%)	0.94	0.89–0.98	0.009
***Initial exposure^e^***					
Within 12 m of diagnosis	211 (0.7%)	913 (0.6%)	1.15	0.98–1.33	0.080
More than 12 m before diagnosis	2,704 (8.6%)	14,694 (9.4%)	0.91	0.87–0.95	<0.001
**DOAC**					
No exposure	30,857 (97.7%)	153,081 (97.6%)	1.00	(*Ref*)	
Ever exposure^b^	551 (2.3%)	2,820 (2.4%)	0.97	0.90–1.06	0.535
Past-use^c^	75 (0.2%)	390 (0.3%)	0.95	0.74–1.22	0.701
Current-use^d^	659 (2.1%)	3,721 (2.1%)	0.98	0.90–1.07	0.598
***Initial exposure^e^***					
Within 12 m of diagnosis	325 (1.0%)	1,687 (1.1%)	0.95	0.84–1.07	0.428
More than 12 m before diagnosis	409 (1.3%)	2,034 (1.3%)	1.00	0.89–1.11	0.898

*OR, odds ratio; CI, confidence intervals; DOAC, direct oral anticoagulants*.

a *matched on age; Adjusted for: T2DM Drugs, Cardiac Drugs, Statins, Charlson Comorbidity Index, Education Level, Marital Status*.

b *defined as two or more prescriptions*.

c *defined as no use within 6 months of PCa diagnosis, two or more prescriptions*.

d *defined as any use within 6 months of PCa diagnosis, two or more prescriptions*.

e *defined as two or more prescriptions, where first prescription is within 12 months of diagnosis, or more than 12 months before diagnosis*.

No association was found between any prior exposure to DOACs and risk of PCa, nor for past or current DOAC use, or timing of initial DOAC exposure (Table 4). Estimates for risk of unfavorable PCa were similar for exposure to warfarin and DOACs (Table 5).

**Table 5:** Adjusted sub-group analyses for risk of favorable and unfavorable prostate cancer.

		**Favorable^a^^,^^b^**			**Unfavorable^a^^,^^c^**	
	**OR**	**95% CI**	***P*-value**	**OR**	**95% CI**	***P*-value**
**Warfarin exposure**						
Never-use	1.00	(*Ref*)		1.00	(*Ref*)	
Ever-use^d^	0.96	0.90–1.02	0.165	0.91	0.85–0.97	0.002
Past-use^e^	0.95	0.86–1.06	0.387	0.84	0.74–0.95	0.005
Current-use^f^	0.96	0.89–1.03	0.254	0.93	0.86–1.00	0.037
***Initial exposure^g^***						
Within 12 m of	1.39	1.13–1.70	0.002	0.98	0.78–1.24	0.894
diagnosis						
More than 12 m	0.93	0.87–0.99	0.027	0.90	0.84–0.96	0.002
before diagnosis						
**Doac exposure**						
Never-use	1.00	(*Ref*)		1.00	(*Ref*)	
Ever-use^d^	1.01	0.90–1.13	0.886	0.94	0.83–1.06	0.326
Past-use^e^	0.92	0.66–1.29	0.647	0.83	0.55–1.26	0.385
Current-use^f^	1.02	0.91–1.15	0.752	0.95	0.83–1.08	0.450
***Initial exposure^g^***						
Within 12 m of	0.95	0.80–1.12	0.529	0.98	0.82–1.17	0.825
diagnosis						
More than 12 m	1.06	0.91–1.23	0.453	0.90	0.76–1.07	0.246
before diagnosis						

*CI, confidence intervals; DOAC, direct oral anticoagulant*.

a *matched on age; Adjusted for: T2DM Drugs, Cardiac Drugs, Statins, Charlson Comorbidity Index, Education Level, Marital Status*.

b *Favorable—low risk (T1–2, Gleason score 2–6 and PSA < 10 ng/ml) and intermediate risk (T1–2, Gleason score 7 and/or PSA 10 to < 20 ng/ml)*.

c *unfavorable—high risk (T3 and/or Gleason score 8–10 and/or PSA 20 to < 50 ng/ml), locally advanced [T4 and/or N1 and/or PSA 50 to < 100 ng/ml in the absence of distant metastases (M0 or Mx)] and distant metastases (M1 and/or PSA ≥ 100 ng/ml and above)*.

d *defined as two or more prescriptions*.

e *defined as no use within 6 months of PCa diagnosis, two or more prescriptions*.

f *defined as any use within 6 months of PCa diagnosis, two or more prescriptions*.

g *defined as two or more prescriptions, where first prescription is within 12 months of diagnosis, or more than 12 months before diagnosis*.

Table 5 displays results of multivariable adjusted analyses for favorable and unfavorable PCa subgroups, with respect to different timing of warfarin and DOAC exposure. These subgroup analyses indicated no association between warfarin use (ever, past, or current) and risk of favorable PCa. In contrast, ever exposure to warfarin was associated with decreased risk of unfavorable PCa (OR 0.91; 95% CI 0.85–0.97). The strongest association for unfavorable PCa was with past warfarin use (OR 0.84; 95% CI 0.74–0.95). Among men with an initial warfarin exposure occurring within 12 months of PCa diagnosis, we observed an increased risk of favorable PCa (OR 1.39; 95% CI 1.13–1.70), but no association with unfavorable PCa (OR 0.98; 95% CI 0.78–1.24). Initial warfarin exposure more than 12 months before diagnosis was associated with slightly decreased risk of both favorable and unfavorable PCa (Table 5).

In contrast to warfarin, no associations were found in relation to ever, past, or current DOAC exposure in either favorable or unfavorable PCa (Table 5). Similarly, no associations were found in relation to timing of initial DOAC exposure in either favorable or unfavorable PCa.

## Discussion

This study found evidence of a small reduction in risk of PCa among men exposed to warfarin compared with those never exposed. This inverse association applied to both past and current users of warfarin. However, the association between warfarin use and overall PCa risk varied depending on the timing of initial exposure, with risk being significantly lower when first exposure was more than 12 months prior to PCa diagnosis, but non-significantly increased when first exposure was within 12 months of diagnosis. The risk of favorable PCa was increased in men who had been exposed to warfarin within 12 months prior to diagnosis. Risk of unfavorable PCa was decreased for all exposures. We found no statistically significant associations between DOAC exposure and risk of PCa, favorable, unfavorable, or overall.

Our finding of decreased PCa risk overall among men exposed to warfarin is consistent with results of several previous studies (7, 10, 12). For example, Tagalakis et al. (12) found that 4 years cumulative warfarin use decreased risk of PCa, though they found no association with ever-use. Following adjustment for age, Pengo et al. (10) found a decreased risk of PCa among men with a history of long-term exposure to VKAs (including warfarin). Similarly, Haaland et al. (7) found a decreased PCa incidence among both warfarin users, and men with atrial fibrillation. However, not all studies have reported a decreased risk among long-term VKA users (5, 6, 8, 9). Blanc-Lapierre et al. (6) found a weak non-significant negative association for oral anticoagulant use and risk of PCa, while Ahern et al. (5) found no evidence of an association for VKA use. However, the former study was based on self-reporting of oral anticoagulant exposure and confounders, which may entail recall bias, and the other identified VKA use by proxy (heart-valve replacement). Kinnunen et al. (8) found a significant increase in PCa risk overall for warfarin ever-use vs. no anticoagulation but no association for 2–5 years cumulative exposure compared with no exposure (8). They also reported increased risk of high grade PCa in men with a history of warfarin use, and metastatic PCa with <2 years of cumulative warfarin exposure. A meta-analysis conducted by Kristensen et al. (9) found no overall association between VKA use and PCa risk (pooled effect estimate of 0.86; 95% CI 0.70–1.05). However, the direction of the pooled effect estimate is consistent with our results.

Taken together, our findings suggest detection bias potentially in two different directions in relation to PCa risk and warfarin exposure. Firstly, a reluctance to biopsy because of an increased risk of bleeding in men on chronic oral anticoagulation represents one possible explanation for an overall reduced risk of PCa (2, 13). Another putative explanation is a perceived decreased life expectancy in men on warfarin leading to a lower diagnostic intensity. On the other hand, the increased risk of favorable PCa among men who recently initiated warfarin treatment suggests there may also be some initial detection bias in the opposite direction. One explanation is that increased consultation rates and routine blood testing required for correct dosing for men starting on warfarin increases the probability of PSA testing (28), leading to increased risk within the first 12 months of exposure. As a consequence, this initial increase in detection of more favorable PCa, could result in a reduced number of PCa cases to be detected, or progress to unfavorable PCa over the next 4–8 years [i.e., equivalent to the lead time for PCa through PSA testing/screening (29)].

It is also possible that the association between warfarin use and PCa risk may be explained by underlying biological mechanisms. Inhibition of the growth arrest-specific 6(Gas6)-AXL tyrosine kinase receptor signaling pathway may provide protection against PCa (30–32). I*n vitro* and *in vivo* evidence suggests that cancer procoagulant (CP), a thrombolytic cysteine protease with direct factor X activating activity, may be vitamin K-dependent and inhibited by warfarin (33). Proposed as an underlying PCa-related coagulopathy (34), CP has been implicated in the metastatic spread of other malignancies *in vivo* (35). CP inhibition by warfarin could explain an overall decrease in unfavorable PCa risk.

Our study found no association between DOAC use and risk of PCa. While our results could reflect a lack of any biological effect of DOACs on PCa risk, it is more plausible that this is due to the relatively shorter exposure time and low number of users within our study participants, given these medications have only recently become available as an alternative anticoagulant therapy. Additionally, differences in point estimates between warfarin or DOAC treatments may be a result of patient selections performed by attending physicians for warfarin or DOAC treatment initiation, due to patient age and number of comorbidities.

Our study has several strengths. PCBaSe 4.0 includes ~98% of all PCa cases in the Swedish Cancer Registry, allowing for a large nationwide study based on high quality detailed information on demographics, drug prescriptions, and comorbidities through linkage to other registers. Information was available to examine possible associations between both warfarin and DOAC exposure and risk groups. Furthermore, we were able to reduce confounding by adjusting for prior use of statins, antidiabetic medications, and cardiovascular medications, which may be associated with risk of PCa and be more commonly prescribed among men using warfarin and DOACs. Individuals with documented venous thromboembolism and similar events were not excluded from this population-based study as they represent a significant population of anticoagulation medication users in contrast to some prior studies (10). Prior cancer diagnoses—a major comorbidity for new disease diagnosis potentially altering the level of thromboembolic events and anticoagulant use—were not an exclusion criterion.

However, several limitations may affect the interpretation of our findings. Use of filled prescriptions may not reflect actual medication use. Because of difficulties in determining the actual medication dose, particularly for warfarin as this is highly individual and variable, we did not consider cumulative or average dose of warfarin or DOACs in these analyses. DOAC's were rarely prescribed prior to and between 2010 and 2016, hence, our study could only investigate relatively short term DOAC exposure and associated PCa risk. Furthermore, no data were available on PSA testing or use of prostate biopsies which precluded identification of diagnostic pathways through which PCa risk may be affected in men using warfarin. Also, data on lifestyle factors such as diet and smoking are unavailable in PCBaSe. In addition, generalisability of our findings may be limited since the population in PCBaSe is predominantly Caucasian with less than ten percent being first generation immigrants.

## Conclusion

We found evidence of decreased risk of Pca in men on warfarin, corroborating results from several previous studies. The decreased overall PCa risk may reflect a reduced biopsy rate among long-term warfarin users due to concerns about bleeding or shorter life expectancy, though this requires further investigation. However, there was an increased risk of favorable PCa in men who recently started to use warfarin, suggesting detection bias due to PSA testing around the start of warfarin treatment. We found no association between use of DOAC and PCa risk, however the number of men on DOACs was limited. Further studies addressing the association between warfarin use and PCa risk should also include data on PSA testing and prostate biopsies among men on anticoagulants.

## Statement of Impact

Several previous studies suggest that risk of prostate cancer may be reduced among men who have been prescribed anticoagulant medications such as warfarin. However, the nature of the association between warfarin and prostate cancer risk remains unclear due to conflicting findings. Newer direct oral anticoagulant medications have been increasingly used in place of warfarin but no studies have examined their association with prostate cancer risk. This population-based study of anticoagulant use found a decreased risk of prostate cancer overall among men exposure to warfarin compared with those never exposed. However, favorable (low-intermediate) prostate cancer risk was increased immediately after commencing on warfarin, while both favorable and unfavorable prostate cancer risk were decreased when initial exposure was more than 12 months earlier, suggesting differing detection biases are operating. No association was found for any exposure to direct oral anticoagulants. Further investigation of prostate specific antigen (PSA) testing and biopsy referral are required to disentangle causal pathways in relation to Warfarin.

## Data Availability Statement

The data analyzed in this study is subject to the following licenses/restrictions: Approval to access data-applies. Data can be accessed from data custodians of NPCR/PCBaSe [www.npcr.se] via an application to the reference group which should be submitted to par.stattin@surgsci.uu.se.

## Ethics Statement

The studies involving human participants were reviewed and approved by Ethical Review Board, Umea University, Sweden. Written informed consent for participation was not required for this study in accordance with the national legislation and the institutional requirements.

## Author Contributions

JA, HG, KB, and MV: conception and design. PS and HG: collection and assembly of data. JP and KB: data analysis. JP, KB, and DC: manuscript writing. JP, KB, DC, HG, BL, PS, JA, ML, JS, and MV: interpretation and critical review and final approval of manuscript. All authors contributed to the article and approved the submitted version.

## Conflict of Interest

The authors declare that the research was conducted in the absence of any commercial or financial relationships that could be construed as a potential conflict of interest.
